# Pathway for inpatients with depressive episode in Flemish psychiatric hospitals: a qualitative study

**DOI:** 10.1186/1752-4458-3-23

**Published:** 2009-10-19

**Authors:** Franciska A Desplenter, Gert M Laekeman, Steven R Simoens

**Affiliations:** 1 Research Centre for Pharmaceutical Care and Pharmaco-economics, Faculty of Pharmaceutical Sciences, Katholieke Universiteit Leuven, ON2 Herestraat 49 PO Box 521, 3000 Leuven, Belgium

## Abstract

**Background:**

Within the context of a biopsychosocial model of the treatment of depressive episodes, a multidisciplinary approach is needed. Clinical pathways have been developed and implemented in hospitals to support multidisciplinary teamwork. The aim of this study is to explore current practice for the treatment of depressive episodes in Flemish psychiatric hospitals. Current practice in different hospitals is studied to get an idea of the similarities (outlined as a pathway) and the differences in the treatment of depressive episodes.

**Methods:**

A convenience sample of 11 Flemish psychiatric hospitals participated in this qualitative study. Semi-structured interviews were conducted with different types of health care professionals (n = 43). The websites of the hospitals were searched for information on their approach to treating depressive episodes.

**Results:**

A flow chart was made including the identified stages of the pathway: pre-admission, admission (observation and treatment), discharge and follow-up care. The characteristics of each stage are described. Although the stages are identified in all hospitals, differences between hospitals on various levels of the pathway exist. Hospitals emphasized the individual approach of each patient. The results point to a biopsychosocial approach to treating depressive episodes.

**Conclusion:**

This study outlined current practice as a pathway for Flemish inpatients with depressive episodes. Within the context of surveillance of quality and quantity of care, this study may encourage hospitals to consider developing clinical pathways.

## Background

According to epidemiological studies, depressive episodes are a burdensome disorder with an important social impact. Depressive episodes are also a common problem in Belgium. Studies estimated the 12-month prevalence at 5-7% and lifetime-prevalence at 15% in the general Belgian population. Furthermore, depressive episode yield an impact on daily life including productivity loss, suicide and reduced social and familial functioning [1]. More and more, the tendency for the treatment of severe and chronic forms of depressive episodes is to use a combined treatment of psychotherapeutic and pharmacological interventions [2,3]. Combined treatments should be based upon a biopsychosocial model of depressive episodes. This conceptual model assumes that psychological (e.g. beliefs) as well as social (e.g. relationships, stress) factors play a role along with the biological factors in human functioning in the context of disease or illness [4,5]. This approach in the treatment of depressive episodes requires interdisciplinary teamwork [6].

Within the context of supporting interdisciplinary teamwork, clinical pathways have been developed and implemented in hospitals [7,8]. Clinical pathways have been defined by Coffey et al. as 'an optimal sequencing and timing of interventions by physicians, nurses and other staff for a particular diagnosis or procedure, designed to minimize delays and resource utilization and to maximize the quality of care' [7]. Developing a clinical pathway is a time-consuming and complex process. The Network Clinical Pathways (Leuven, Belgium) developed a 30-steps plan to guide teams in their development and implementation of such clinical pathways. The main purpose of this Network is to support Belgian and Dutch hospitals to develop, implement and evaluate clinical pathways by providing education and support to hospitals and by conducting research on clinical pathways [9,10]. The 30-steps plan comprises four phases based on the quality cycle of Deming [11]. Phase one, the planning phase, comprises six steps focussing on the composition of a multidisciplinary team, selection of the patient population and first version of the pathway according to current practice. Phase two, the doing phase, comprises seven steps focussing on the collection of data on current practice and best practice. Phase three, the checking phase, comprises seven steps focussing on the evaluation of collected data and improvement of the pathway. Phase four, the acting phase, comprises ten steps focussing on the implementation and the continuous evaluation of the pathway [12].

Benefits have been documented on the use of clinical pathways. These benefits include reduced length of stay, reduced costs and improved communication between the members of the multidisciplinary team [7]. To date, clinical pathway development and use has been limited in mental health care settings as compared with medical and surgical care settings [13]. Barriers to the implementation of clinical pathways in mental health care include the difficulty in defining the nature, patient individuality and variability of mental illness [14-16]. Evans-Lacko et al. reviewed eight studies on the impact of clinical pathways in mental health. The overall scientific quality was poor and the results of the studies were mixed [13].

The overall aim of this study was to explore current practice for the treatment of depressive episodes in Flemish psychiatric hospitals. The specific objective of this study was to describe the pathway followed by an inpatient with a depressive episode. A clinical pathway is specific to one hospital. This study wants to explore current pathways of different hospitals in order to get an idea of the similarities and differences in the treatment of depressive episodes in Flemish psychiatric hospitals.

## Methods

### Setting

A convenience sample of 11 Flemish psychiatric hospitals participated in this study during the period April 2007 - September 2008. Hospitals were asked to participate via the Flemish Association of Psychiatric Hospital Pharmacists (VZA-Psychiatry). In Flanders, most hospitals are part of a network of hospitals. Our sample included individual hospitals as well as hospitals from different networks: 'Broeders van Liefde', 'Broeders Hiëronymieten' and university hospitals. The hospitals are located in different geographical regions of Flanders. The number of beds per hospital ranges from 85 to 500 (mean ± standard deviation: 214 ± 106). All hospitals treated inpatients with a depressive episode. Approval of the hospital management to perform the study was obtained for all hospitals. This study was part of a larger study on the treatment of depressive episodes for which ethical approval in the 11 hospitals was obtained.

### Data collection

Two main data sources were used. First, the websites of the 11 psychiatric hospitals were searched for available information on their approach to treating depressive episodes [17-27]. Second, health care professionals of the 11 hospitals were interviewed in a semi-structured way. *In vivo *interviews were chosen as this study wants to explore current practice. A purposive sample of health care professionals dealing with inpatients suffering from mood disorders was interviewed. Health care professionals eligible for the interview included psychiatrists, nurses, pharmacists, psychologists, a discharge manager as well as a patient care manager working on a ward for mood disorders. A list of eligible health care professionals was compiled for every hospital by the hospital management and hospital pharmacist. The listed health care professionals were contacted by telephone by the main researcher (F.D.) to explain the purpose of this study and to invite them to participate. The interview guide included the following topics: admission, therapy, evaluation and discharge. For each topic, aspects of time, content, methods, influencing factors, involved health care professionals, offered treatment and follow-up care were discussed. A basic scheme with the main stages of a hospital stay was discussed and refined. The interview ended with the question if all aspects of the hospital stay for depressive episodes were discussed and if there was anything else the interviewee wanted to add in order to have a factual picture of a hospital stay for depressive episodes. Demographic characteristics of the interviewees were collected. The interviews were audio-taped and additional notes were made during the interviews. Interviews and notes were handled anonymously. Some health care professionals spontaneously provided written documents on the activities and the ward-specific goals of treatment of mood disorders.

### Data analysis

The main researcher (F.D.) completed reports by listening to the tape recorded interviews. Stages of hospital stay and their characteristics were identified inductively. A pathway was designed for each hospital based on the website information and the interviews. If some information was unclear or lacking, the appropriate health care professional was mailed or telephoned by the main researcher (F.D.) to solve this deficiency. Finally, the 11 pathways were analysed inductively for commonalities and differences. These commonalities were the basis in order to trace out a general pathway for the treatment of depressive episodes.

Quotes were selected with respect to the different types of health care professionals of the participating hospitals.

## Results

Forty-three health care professionals from 11 Flemish psychiatric hospitals consented to be interviewed. An overview of the interviewed health care professionals per hospital can be found in Table 1. One to eight interviews were performed in each hospital. The mean duration of the interviews was 21 minutes (standard deviation of 8 minutes) with a range from 10 till 35 minutes. Twenty-one of the interviewees were men. The median year of graduation was 1992 (range: 1971-2005). They had a mean of 16,31 ± 9,45 years of experience in psychiatry (range: 2-36 years) and a mean of 9,53 ± 8,20 years of experience in their current position in the hospital (range: 0,5 - 33 years). Quotes are referenced to the number of the hospital (1-11; see Table 1), the kind of interviewee (D = doctor, psychiatrist/(H)N = (head) nurse/P = pharmacist/Y = psychologist/DM = discharge manager/PCM = patient care manager) and a number if more than one person of the same type was interviewed (1-3) within the same hospital.

**Table 1:** Overview of the interviewees in each hospital (H)

**Interviewees**	**H1**	**H2**	**H3**	**H4**	**H5**	**H6**	**H7**	**H8**	**H9**	**H10**	**H11**	**Total**
**Doctor/psychiatrist**	1	-	1	3	1	3	-	1	2	2	1	**15**

**(Head) nurse**	2	1	2	3	2	3	1	1	3	1	-	**19**

**Pharmacist**	-	-	-	-	-	-	-	-	-	-	1	**1**

**Psychologist**	-	-	1	-	2	2	-	-	-	1	-	**6**

**Discharge manager**	-	-	-	-	-	-	-	-	-	-	1	**1**

**Patient care manager**	-	-	-	-	-	-	-	-	-	-	1	**1**

**Total**	**3**	**1**	**4**	**6**	**5**	**8**	**1**	**2**	**5**	**4**	**4**	**43**

**Figure 1:**
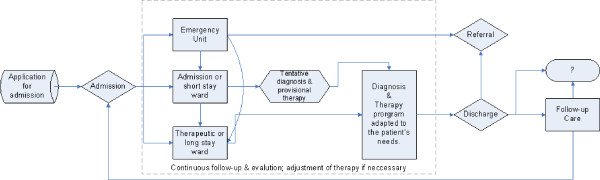
**Flow chart of the pathway for depressive episode in a psychiatric hospital**. The ? refers to the unclear continuation of the pathway.

Based on the interviews and on the information available on the website of the hospital, a general flow chart was constructed (see Figure 1). This flow chart includes the different stages of the pathway that were identified. It started from the application for admission, also called the pre-admission stage. If the decision was made to admit a patient, he/she could be admitted to different types of wards. Diagnosis and therapy were the two main parts of this 'admission' stage. At an unspecified point of time, the patient was discharged. The patient could be referred to another institution, to other health care professionals or to ambulatory care. Follow-up care could be organized. If needed, patients could be readmitted. In some cases, the continuation of the pathway is unclear e.g. in cases of discharge against authority (this is when a patient discharged him/herself from hospital against the medical advice of the multidisciplinary team).

8HN: Patients can be admitted through various ways: referral by the general practitioner, referral by another hospital, on demand of the patient him/herself, ...

11D: If you talk about follow-up care, you certainly have to talk about pre-care. This is the application of the patient to be admitted to the hospital. This is done by a prior talk. Afterwards, the patient is admitted or is not admitted to the hospital.

All hospitals had a specific ward or at least a specific group within a ward for patients with mood disorders. This was an acute (short stay) ward or a chronic or therapeutic (long stay) ward. According to the health care professionals interviewed, patients could be admitted directly to either of these two types of wards. The different stages of the clinical pathway for depressive episodes as described above were pre-admission, observation/diagnostics, treatment, discharge and follow-up care. The following characteristics of each stage are described [see Additional file 1]: treatment aim, approach, extent of patient's responsibility, setting, content, influencing factors, involved health care professionals, evaluation, follow-up and duration.

11DM: I think the multidisciplinary team of health care professionals as a whole is involved when a patient is admitted: the psychiatrist as well as the psychiatric nurse, the social worker, the occupational therapist, the psychologist euh ... the individual counselor. Everyone has his/her own role in this process and performs that role from his/her professional point of view.

2HN: The general aims of the ward will be translated into individual aims in consultation with the patient and the team of health care professionals. This search is done through an open dialogue and with shared responsibility. The patient and his/her aims are evaluated monthly in the team meetings.

Therapy programs in the hospitals contained the cornerstones of the biopsychosocial model of health and illness[4,5] Health care professionals reported that therapy programs were on the one hand adapted to the patient's aims of admission and on the other hand adapted to patient-specific factors like skills, intellectual properties, social environment, etc. Every patient was said to be assigned to an individual counsellor who guides the patient during his/her hospital stay. Health care professionals reported that the preparation to discharge the patient starts from the moment of admission, considering the patient's background and daily environment.

11V1: It is mainly a therapeutic program for groups with individual emphases. It is based on four models: cognitive behavioral therapy, system therapy, euh activation therapy and body-oriented therapy.

11Y: Psycho-education, that's information on anxiety and depression ... that is really a course.

4N1: So, every patient receives an individual counsellor throughout the entire hospital stay.

10D1: From day one of admission, discharge will be prepared. If a patient does not a have a social network, we shall be attentive that the hospital stay will not be an isolated process running the risk that the patient will be fully isolated from day one after discharge.

Although participants reported applying a variety of methods in the follow-up of patients, four approaches came up in all hospitals. The first approach was subjective: what does the patient tell? The second approach was objective: what does the team observe? The third approach included an evaluation of the patient: are there any changes during the admission of the patient - via observation or via measurements? The fourth approach included a discussion of the planning: how does the planning proceed and is there a need to adjust the planning?

10D2: The nurses keep seeing the patient every day.

7HN: The patient can only proceed to the next stage if they fulfill all criteria. That's the reason why there may be some variation in the duration of every stage.

11D: Within the ward, we use a model. First, you evaluate subjectively: what does the patient tell, which are his/her complaints? 'I have a headache.' 'I feel dispirited.' 'I have no appetite.' ... Then, you have the evaluation of how we look at this objectively. For example ... he has no appetite but he weighs 80 kilograms for his 1.70 meter ... or 40 kilograms for 1.80 meter ... that's also possible. Euh, then we have the evolution: how do these things change during the hospital stay? And of course programming and planning. For each problem, you can have such an evaluation. ... For example: relational problems. Subjective: the patient reports relational problems. Objective: how do you assess this? The couple is arguing. Evaluation: OK, let's do something about it and then you have to follow-up if the partners are coming back together or not or if they start al least talking again to each other ...

The above results described the common approach of the hospitals studied. Some differences were observed between the hospitals. First, the reported members of the multidisciplinary teams differed, e.g. pharmacists did not systematically belong to the team. Second, specific medical treatment for depressive episodes, e.g. electroshock therapy, was only available in specialized hospitals. Third, duration of therapy and hospital stay was fixed in some hospitals, but was flexible in other hospitals. Duration for the treatment of depressive episodes differed between patients according to the complexity of the problem but also differed between hospitals. Differences in the duration of the different stages were observed between hospitals. Fourth, psychotherapeutic programs and non-medical programs differed according to the facilities of the hospital. Fifth, every hospital said that it evaluated patients on a regular basis, even though the frequency of evaluation could vary. Sixth, scales were used during follow-up and evaluation of the patient, but the amount and type of scales reported by the participants differed between hospitals. Seventh, communication to the patient's family or relatives was said to be incorporated in the process of hospital stay or was said to be performed on demand.

10V1: At our hospital, the patient is discharged after 13 weeks in any case ... the patients do know this. ... Afterwards, patients can be referred ... to ambulatory care, an outpatient mental health centre or another hospital.

11DM: Oh, ... indeed, it does happen that at certain points, the family is involved in the process but not every patient has a social environment to count on. There are patients who say: 'please, leave my family out of this' ... so, you have to have respect for the wishes of the patient.

## Discussion

The study identified the different stages of a hospital stay for the treatment of depressive episodes which were present in all eleven hospitals. These were: pre-admission or orientation, admission consisting of observation/diagnostics and treatment, discharge and follow-up care. For each stage the aim, patients' extent of responsibility, approach, setting, content, influencing factors, involved health care professionals, evaluation/follow-up and duration were described. However, there were some differences between hospitals. The above results described the general current practice which is offered in the selected hospitals. Each hospital emphasized that each patient is approached on an individual basis. Although one hospital wanted to start the development of a clinical pathway, none of the studied hospitals used clinical pathways during this study.

As we wanted to learn about current practice and approach of the treatment of depressive episodes, we chose a qualitative methodology. This methodology is suitable to learn more in-depth about procedures employed in the psychiatric hospitals [28]. Our focus was on how the treatment process for depressive episodes was managed.

Clinical pathways are especially relevant for patients who have complex problems as these pathways allow for the integration of interventions of the multidisciplinary team which is needed in such cases. This may improve the coordination and quality of care. During the interviews, health care professionals emphasized the patient-focused approach. Patient individuality and variability is an important barrier to the development and implementation of clinical pathways which have been reported in previous studies in Australia and the USA [14,15,29]. The continuous follow-up and evaluation of patients may result in adjustments of the treatment complying with the issue of individuality and variability of patients.

We identified five stages in the hospital stay for depressive episodes: from pre-admission till follow-up care. The first four stages were also identified in an Australian study [29]. Follow-up care in the period after discharge was identified as a fifth stage. This result is endorsed by Bultema et al. as they suggested that clinical pathways need to include the pre-admission and post-discharge phases. Clinical pathways should be designed for a variety of diagnoses across the care continuum [30].

Therapy programs of all hospitals comprised a combination of approaches: pharmacological as well as psychotherapeutic interventions and focus on social aspects. In the discharge process, attention is paid to rehabilitation and prevention of relapse. If desirable, follow-up care is organized. This combined approach is concordant with the biopsychosocial model of depression and with current guidelines for the management of depression [2]. As mental healthcare is complex, mental health clinical pathways will have to remain flexible and allow for the individuality and specificity of the patient and his/her environment [29].

Our approach has several strengths. The sources of information used were two-fold: internet information and semi-structured interviews. If possible, several health care professionals of one hospital were interviewed. This corroborates the convergence of the results as well as the different aspects of the biopsychosocial model [4,5]. Moreover, attempts were undertaken to have a clear and complete picture of every hospital. A convenience sample of several types of health care professionals was therefore applied. This sample was based on a list of eligible health care professionals compiled by the hospital management and hospital pharmacist. This approach may potentially have limited the number and types of health care professionals included in this study. To ensure a complete picture of every hospital, the appropriate health care professionals were contacted in case of deficient data on a certain theme. To achieve an overall saturation on all themes, probably more interviews should have been conducted. Additionally, eleven psychiatric hospitals were involved in our study, representing about one third of all psychiatric hospitals in Flanders. This may provide an approximation of the approach in Flanders for the treatment of inpatients with major depression. To the best knowledge of the authors of this article, this is the first time a pathway is described of current practices in multiple hospitals, even though there are multiple publications on developed and implemented pathways in mental health care [13-16,29,30].

There are some limitations to our approach. Regarding the methodology of the development of a clinical pathway, our study was limited to only one step (phase 'plan') of the 30-steps plan which was described in the introduction section [12]. Data collection was done by semi-structured interviews on a one-to-one basis and not within a multidisciplinary team, as normally done for clinical path development. However, different types of health care professionals were invited to participate in an interview. In most hospitals several types of health care professionals were interviewed. By doing so, the different points of view of the biopsychosocial model have been considered [4,5].

Another structural limitation is the lack of a daily schedule as this is commonly used in clinical pathways (e.g. in Bultema et al. 1996) [30]. The treatment duration is variable from weeks till months according to the severity and complexity of the patient's mental health status. The evolution of the patient is unpredictable. As a consequence, it is very difficult to develop such daily schemes in the context of a clinical pathway [14,16].

In our study, we selected a patient population with depressive episodes. The results of this study can not be generalized to other populations in mental health and to other settings. The methods used and the identified stages can be a framework for hospitals which would like to develop a clinical pathway. The selection of a specific patient population is intrinsic to the development and implementation of a clinical pathway [12].

Finally, we did not make any comparison of the reported treatment process with real patient files in order to check for convergence. However, the data collected in our interviews pointed to an *ad hoc *and individual approach of each patient.

According to the Australian experiences of Emmerson et al., the pathway development within the multidisciplinary team was as crucial as the completed pathway itself. Therefore, they suggest that implementation should only been done when the pathway has been locally developed [29]. The pathway outlined in this study gives an overview of current practice for depressive episodes in Flemish psychiatric hospitals. If hospitals wish to implement a clinical pathway for depressive episodes in their hospital, the methods used and the described pathway can be of assistance to the design of their pathway of current practice. The whole process of developing and implementing a clinical pathway for a specific patient population should be done separately for each hospital.

The question remains if mental health clinical pathways in practice really contribute to the quality and the quantity of provided care. Further studies are needed to provide evidence on the effectiveness and cost-effectiveness of mental health clinical pathways before we can know if they may contribute toward a better mental healthcare [13].

## Conclusion

This study outlined current practice as a pathway for inpatients with depressive episodes in Flemish psychiatric hospitals. Although, the main stages were identified in all hospitals, differences on various levels of the pathway exist. Each hospital emphasized the individual approach of each patient. With this study, we hope to make hospitals aware of the importance of the quality and quantity of the provided care. Therefore, we encourage hospitals to consider developing clinical pathways in their own hospital with their own multidisciplinary team.

## Competing interests

The authors declare that they have no competing interests.

## Authors' contributions

FD performed the design of the study, participated in the data collection, carried out the data analysis and drafted the manuscript. GL and SS participated in the design of the study, supervised the study and redrafted the manuscript. All authors read and approved the final manuscript.

## Supplementary Material

Additional file 1 **Characteristics of the different stages of the clinical pathway for depressive episodes**. This table gives an overview of the characteristics of the different stages of the clinical pathway for depressive episodes. Click here for file
