# Supplementary material for: Pathway for inpatients with depressive episode in Flemish psychiatric hospitals: a qualitative study
Source: Int J Ment Health Syst. 2009 Oct 19;3:23. doi: 10.1186/1752-4458-3-23 (PMC2770989; doi:10.1186/1752-4458-3-23)
Supplement: Additional file 1 — Characteristics of the different stages of the clinical pathway for depressive episodes. This table gives an overview of the characteristics of the different stages of the clinical pathway for depressive episodes. [file 1752-4458-3-23-S1.DOC]

| **Stages** | **Orientation**  **= pre-admission** | **Admission:**  **Observation and Diagnostics** | **Admission:**  **Treatment** | **Discharge** | **Follow-up care** |
| --- | --- | --- | --- | --- | --- |
| **Aim** | Assessment of need for admission | Supportive and understanding care.  To let patients calm down. | Structuring  Remission of depression | Rehabilitation and prevention of relapse | Coping with arising depressive symptoms.  Prevention of relapse. |
| **Extent of patient’s responsibility** | Motivation for admission | Minimal expectations towards patient’s responsibility | Encouragement/appeal of patient’s responsibility | Strong emphasis on patient’s responsibility | Strong emphasis on patient’s responsibility |
| **Approach** | Intake talk,  introductory interview | Bio-psycho-social model  Contextual – phenomenological – holistic. | Bio-psycho-social model  Contextual – phenomenological – holistic. | Bio-psycho-social model  Contextual – phenomenological – holistic. | According to the choice of follow-up care. |
| **Setting** | Background setting of patient:  - home - day clinic - assisted living - general hospital - psychiatric hospital - nursing home  Referral by: - general practitioner - psychiatrist - mental health centre - psychologist - patient him/herself - family or relatives - forced admission. | - Emergency unit  - Acute or admission ward (open or closed) – (short stay)  - Therapeutic ward (long stay) | - Acute or admission ward (short stay)  - Therapeutic ward (long stay) | Setting after discharge:  - home - part-time admission - assisted living - (psychiatric) nursing home - (psychiatric) hospital - homeless home | Follow-up care possibilities:  - day clinic  - rehabilitation after-care  - mental health centres  - ambulatory care (psychiatrist, psychologist, general practitioner, …) - forced follow-up care - psychiatric homecare |
| **Content** | Patient profile:  - first admission or readmission - aims of admission - psychiatric anamnesis including suicidal characteristics - major events in personal life | Crisis stabilization  Get to know each other  Tentative diagnosis based on:  - observation in all disciplines - consultation psychiatrist (DSM-IV, diagnostic scales, screening, …) - talks with responsible nurse  - assessment of personal characteristics - psychological, fysical and neurological examination - if needed: consultation psychologist and/or social worker - if needed: consultation with relatives  Provisional therapy program  Formulate aims  In search of appropriate pharmacotherapy | ‘Confirmation’ diagnosis  Therapy program adapted to patient’s needs and aims. Including group and individual sessions.  - pharmacotherapy - medical psychiatric consultations  - cognitive behavioral therapy - system therapy - activation therapy - body-oriented therapy - psychomotor therapy - psycho-analysis - communication skills - contacts with responsible nurse  - family therapy - activities of daily life - sports, cooking, music, relaxation, creativity - life on the ward | Planning of discharge and follow-up care:  - re-establish social contacts - guidance to resumption of work - (re)orientate living situation - prevention of relapse - social reintegration - plan for the day - encourage compliance  Therapy program: see column on the left. | Adapted to the patient’s needs and mental health condition.  - long-term pharmacotherapy in order to prevent relapses  - psychotherapy - consultations |
| **Influencing factors** | - Waiting list  - Urgency  - Motivation of patient | - Patient-specific problems, aims and preferences  - Patient profile: IQ, personality, …  - Stage ‘observation and diagnostics’ can be omitted when diagnosis is already known. | - Patient-specific problems, aims and preferences  - Patient profile: IQ, personality, …  - Available therapy options on the ward | - discharge against authority: discharge planning is not possible - patient’s mental health condition - patient’s preferences - discharge setting / caring environment | - patient’s mental health condition - patient’s preferences - availability of follow-up care - discharge setting / caring environment |
| **Involved health care professionals** | - Admission coordinator  - Psychiatrist  - Psychologist  - Charge nurse | Multidisciplinary team | Multidisciplinary team:  supervisor, psychiatrist, psychologist, nurses, social worker, therapist (ergo, psychomotor, creativity, family, …) | Multidisciplinary team  Social services | According to the choice of follow-up care. |
| **Evaluation and follow-up** | Decision on admission by limited number of team-members. Choice of hospital ward. | Multidisciplinary team meetings at regular times. Mini team meetings. Daily staff briefings. Patient medical file.  Diagnostics based on observation, scale measurements and talks/consultations with the patient. | Multidisciplinary team meetings at regular times. Mini team meetings. Daily staff briefings. Patient medical file.  Evaluation based on observation, scale measurements, talks/consultations with the patient, realization of aims.  Adjustments to therapy program if necessary.  Feedback to patients on their progress. | Subjective appraisal:  - aims realized? - patient sufficiently recovered? - social context ready?  - follow-up care arranged? | According to the choice of follow-up care. |
| **Duration** | 1 or multiple talks | Hours – Days – Weeks (1-4) | Several weeks – months (maximum 1 year) according to the complexity of the patient’s condition. | Starts at time of admission.  Focus on rehabilitation and prevention of relapses in last weeks of admission. | None – several months – several years |
